# Resident CD11b^+^Ly6C^−^ Lung Dendritic Cells Are Responsible for Allergic Airway Sensitization to House Dust Mite in Mice

**DOI:** 10.1371/journal.pone.0053242

**Published:** 2012-12-31

**Authors:** Claire Mesnil, Catherine M. Sabatel, Thomas Marichal, Marie Toussaint, Didier Cataldo, Pierre-Vincent Drion, Pierre Lekeux, Fabrice Bureau, Christophe J. Desmet

**Affiliations:** 1 Laboratory of Cellular and Molecular Immunology, GIGA-Research and Faculty of Veterinary Medicine, University of Liège, Liège, Belgium; 2 Laboratory of Tumours and Developmental Biology, GIGA-Research, University of Liège, Liège, Belgium; 3 Laboratory of Preclinical and Biomedical Sciences, University Hospital Center, University of Liège, Liège, Belgium; French National Centre for Scientific Research, France

## Abstract

Conventional dendritic cells (DCs) are considered to be the prime initiators of airway allergy. Yet, it remains unclear whether specific DC subsets are preferentially involved in allergic airway sensitization. Here, we systematically assessed the respective pro-allergic potential of individually sorted lung DC subsets isolated from house dust mite antigen (HDM)-treated donor mice, following transfer to naïve recipients. Transfer of lung CD11c^+^CD11b^+^ DCs, but not CD11c^+^CD11b^−^CD103^+^ DCs, was sufficient to prime airway allergy. The CD11c^+^CD11b^+^ DC subpopulation was composed of CD11c^+^CD11b^+^Ly6C^+^ inflammatory monocyte-derived cells, whose numbers increase in the lungs following HDM exposure, and of CD11c^+^CD11b^+^Ly6C^−^ DCs, which remain stable. Counterintuitively, only CD11c^+^CD11b^+^Ly6C^−^ DCs, and not CD11c^+^CD11b^+^Ly6C^+^ DCs, were able to convey antigen to the lymph nodes and induce adaptive T cell responses and subsequent airway allergy. Our results thus support that lung resident non-inflammatory CD11c^+^CD11b^+^Ly6C^−^ DCs are the essential inducers of allergic airway sensitization to the common aeroallergen HDM in mice.

## Introduction

Because it is key to the understanding, prevention and treatment of airway allergy, the question of how inhaled allergens lead to the activation of T_H_2 responses, the major orchestrators of allergy [1], is a subject of intense investigation. Over the last decade, a strong case has been built for a model in which lung conventional dendritic cells (DCs), the most "professional" lung antigen presenting cells, act as antigen-sampling sentinels responsible for the initial activation of T_H_2 cells [2], [3]. Indeed, DCs are present throughout the respiratory system as a network of immune cells that rapidly take up inhaled antigens and convey them to the lung draining lymph nodes where they may activate antigen-specific T cell responses [4]. The notion that DCs play a seminal role in allergic airway sensitization received support from ablation studies using transgenic mice expressing the diphteria toxin receptor (DTR) under the dependence of the *Cd11c* promoter (CD11c-DTR mice), in which administration of diphteria toxin allows for the depletion of DCs in the lung and lung draining lymph nodes. Diphteria toxin-treated CD11c-DTR mice indeed are impaired in their ability to mount T_H_2 responses and airway allergy in models of allergic airway sensitization to house dust mite antigens (HDM), the major respiratory allergens in humans [5]. Adoptive transfer experiments have also been extensively used as an alternative to ablation approaches to support a role for DCs in allergic airway sensitization. Classically, in these experiments, DCs isolated from the spleen of naïve mice [6] or derived *in vitro* from bone marrow progenitor cells [7] are loaded with antigen through *in vitro* culture, and transferred to naïve recipient mice, in which they induce antigen-specific T_H_2 responses.

Lung DCs are now known to represent a heterogeneous cell population [3]. In the steady-state, lung DCs comprise CD11c^+^CD11b^−^CD103^+^ DCs and CD11c^+^CD11b^+^ DCs [8], [9] and upon allergen exposure, the pool of CD11c^+^CD11b^+^ DCs expands due to the recruitment of inflammatory DCs, which are thought to derive mainly from blood Ly6C^+^ monocytes [5], [10]. Recent evidence supports that these lung DC subsets may have specialized functions, for instance in the induction of adaptive antiviral responses [2], [3].

Whether a specific subset of lung DCs is involved in allergic airway sensitization however remains unclear, as recent studies yielded contradictory conclusions. In a first report, Hammad et al. [5] principally ruled out a major role of basophils as antigen-presenting cells in the development of allergen-specific T_H_2 cells, but also proposed that inflammatory DCs instead are able to induce airway allergy to HDM. The authors aimed at demonstrating this latter conclusion *in vivo* by combining two approaches. First, they showed that intraperitoneal (i.p.) adoptive transfer of total DCs or FcεRI^+^DX5^−^ cells (containing approximately 75% of CD11c^+^ DCs) isolated from the lung draining lymph nodes of HDM-treated donor mice is sufficient to induce allergic sensitization in naïve recipient mice. As a second line of evidence, they observed that allergic airway sensitization may be transferred by bone marrow precursors differentiated into bone marrow-derived DCs (BMDCs) by GM-CSF (granulocyte macrophage-colony stimulating factor) treatment, which supposedly resemble inflammatory DCs, but not by BMDCs derived from precursors cultured with Flt3-ligand (Fms like tyrosine kinase 3 ligand), which supposedly more closely resemble steady-state DCs [11]. As a cautionary note yet, a very recent study suggests that this concept should be revisited as it showed that inflammatory DCs in the lung develop independently of GM-CSF, whereas this cytokine is involved in the development and homeostasis of non-inflammatory resident CD11b^−^CD103^+^ and CD11b^+^ DCs [12]. In contrast with a scenario involving inflammatory DCs, a second recent study proposed that CD11b^−^CD103^+^ DCs instead are involved in allergic airway sensitization, whereas CD11b^+^ DCs in general would only be able to prime allergen-specific T_H_1 responses [13]. To support this conclusion, Nakano et al. observed that naïve T cells cultured with CD11b^−^CD103^+^ DCs derived from the lung or draining lymph node of allergen-exposed mice preferentially produce T_H_2 cytokines following activation with phorbol myristate acetate and ionomycin or with anti-CD3 and –CD28 agonistic antibodies. T cells cocultured in the same conditions with CD11b^+^ DCs in contrast produced mainly T_H_1 cytokines [13]. To support that CD11b^−^CD103^+^ DCs indeed may prime airway allergy *in vivo*, the same authors reported that BXH2 mice, which lack CD103^+^ DCs due to a point mutation in the interferon (IFN) regulatory factor (*Irf*)*8* gene [14], are protected from airway allergy induced by endotoxin-adjuvanted ovalbumin (OVA) and house dust extracts [13]. However, BXH2 mice also lack CD8α^+^ lymph node DCs [14], and present additional immunological alterations [15], [16].

Clearly, notwithstanding the advances they allowed or suggested, current ablation and genetic deficiency models have significant limitations when it comes to the identification of lung DC subsets involved in allergic airway sensitization. Indeed, ablation studies in DTR transgenic mice only allow the depletion of DCs at once in the lung and the lung draining lymph nodes and affect multiple DC subsets [17], [18]. They may furthermore lead to DC-unrelated effects [19], [20]. Similar limitations apply to models of genetic deficiency in given transcription factors [14].

Although adoptive transfer studies may offer a valid alternative to ablation studies, most past attempts used spleen and bone marrow-derived DCs, which are only handy surrogates for lung DCs and may have a very different biology. Here, using a model of adoptive transfer of lung DCs isolated from HDM-exposed mice, we studied the pro-allergic activity of the different subsets of lung cells classically identified as lung DCs in a systematic dichotomic approach.

## Materials and Methods

### Mice

Wild-type CD45.1 and CD45.2 C57Bl/6 mice were from The Jackson Laboratory (Bar Harbor, ME). All mice were housed and bred in our specific pathogen free facility. Females were used at 8–10 weeks of age. All experiments were conducted with approval of the Institutional Animal Care and Use Committee of the University of Liège. We also followed the "Guide for the Care and Use of Laboratory Animals" prepared by the Institute of Laboratory Animal Resources, National Research Council, and published by the National Academy Press (revised 1996).

### Reagents and Abs

Lyophilized HDM (*Dermatophagoides pteronyssinus*) extracts were from Greer Laboratories (Lenoir, NC). OVA-fluorescein isothiocyanate (FITC) was from Invitrogen (Life Technologies Ltd., Paisley, Scotland). Methacholine was from Sigma-Aldrich (St Louis, MO). Allophycocyanin (APC)-eFluor780-conjugated anti-CD11c (N418), biotinylated anti-CD40 (1C10), biotinylated anti-CD86 (GL1), biotinylated anti-CD80 (16-10A1), biotinylated anti-FcεRI α (MAR-1), biotinylated anti-F4/80 (BM8), biotinylated anti-CCR7 (4B12), biotinylated anti-Jagged-2 (HMJ2-1), biotinylated anti-CD8α (53-6.7), Phycoerythrin(PE)-conjugated anti-ICOS-L (HK5.3), PE-conjugated anti-Jagged-1 (HMJ1-29) and PE-conjugated anti-TIM-4 (RMT4-54) were from eBioscience (San Diego, CA). 7-aminoactinomycin (7-AAD), FITC-conjugated anti-CD103 (M290), V450-conjugated anti-CD11b (M1/70), FITC-conjugated anti-Ly6C (AL-21), biotinylated anti**-**MHC class II (I-A^b^) (AF6-120.1), APC-conjugated anti-SIRPα (P84), APC-conjugated anti-Ly6C (AL-21), APC-conjugated anti-CD117 (c-kit; 2B8), biotinylated anti-OX40L (RM134L) and APC-streptavidin were from BD Biosciences (Mississauga, Ontario, Canada). Isotype controls and antibodies were from the same manufacturer. 2.4G2 Fc receptor antibodies were produced in house.

### Phenotypic Characterization and Isolation of Lung DC Subsets

Lightly isoflurane-anesthetized mice received an intranasal (i.n.) instillation of vehicle (endotoxin-free saline) or HDM extract (100 µg in 50 µl). Single cell suspensions from lungs and bronchial lymph nodes (BLNs) were obtained 12, 24 or 48 hours (h) later as described before [21], incubated at 4°C with 2.4G2 Fc receptor antibodies to reduce non-specific binding and then with conjugated antibodies. Flow cytometry was performed on a FACScanto II (Becton Dickinson, Mountain View, CA). In experiments aimed at isolating DC subsets, cell suspensions were first enriched in DCs by MACS using CD11c MicroBeads (Miltenyi Biotec, Bergisch Gladbach, Germany) and then sorted on a FACSAria II (Becton Dickinson). Alveolar macrophages were excluded based on their autofluorescence and CD11c^hi^ phenotype. Cell viability was always assessed using 7-AAD. The purity of sorted DC populations always was >95%.

### Mouse Model of Allergic Airway Sensitization by Adoptive Transfer of Lung DCs

Unless specified otherwise, 1×10^6^ FACS-sorted lung DCs from C57BL/6 mice that were injected i.n. 24 h earlier with either 100 µg HDM or saline were transferred into the trachea of anesthetized naive recipients. Ten and 11 days (d) later, recipient mice were challenged with 5 µg HDM i.n. Airway hyperresponsiveness (AHR) and airway allergy were evaluated 3 d later. Alternatively, i) CD45.1 rather than CD45.2 recipients were used, and ii) 3×10^4^ FACS-sorted BLN DCs from C57BL/6 donor mice were injected i.p. to naive recipients.

In experiments aimed at determining the requirement for *in vivo* HDM exposure, lung CD11c^+^ DCs were sorted from mice treated 24 h earlier with HDM or saline, and cultured in the presence or absence of HDM (30 µg/ml) for 18 h in RPMI 1640 medium supplemented with 10% FBS, 2 mM L-glutamine, 1 mM sodium pyruvate, 0.1 mM non-essential amino acids, 50 µM β-mercaptoethanol, 50 µg/ml streptomycin, and 50 IU/ml penicillin (all from Gibco-Invitrogen). 5×10^5^ live cultured lung DCs were then transferred to naïve recipients that were challenged and analyzed as described above.

### Assessment of Airway Allergy

Bronchoalveolar lavage (BAL), BAL cytology and lung histology were performed as described previously [21]. AHR was estimated by assessing dynamic airway resistance in anesthetized animals using a FlexiVent small animal ventilator (SCIREQ), as previously described [21]. Changes were expressed as percentage change from the baseline measurement (performed after PBS exposure). BLN cells were collected and restimulated as previously reported [22]. Supernatants of BLN cells were assayed for interleukin (IL)-4, IL-5, IL-13, IL-17 and interferon-γ (IFN-γ) by ELISA (eBioscience [IL-4 and IFN-γ]; Invitrogen [IL-5 and IL-13]; Biolegend [IL-17]). ELISA sensitivities were as follows: IL-4<5 pg/ml; IL-5<3 pg/ml; IL-13<2 pg/ml; IL-17<2.7 pg/ml; IFNγ <1pg/ml.

### DC Migration

To assess lung DC migration, mice were injected intratracheally (i.t.) with either 50 µg HDM admixed to 100 µg OVA-FITC or saline. Twelve and 24 h later, lungs and BLNs were analyzed by flow cytometry for the presence of antigen-loaded DCs (FITC^+^ DCs).

### Measurement of Immunoregulatory Cytokines

CD11b^+^Ly6C^+^ and CD11b^+^Ly6C^−^ lung DCs were isolated from HDM-treated mice and cultured overnight in RPMI 1640 medium supplemented with 10% FBS, 2 mM L-glutamine, 1 mM sodium pyruvate, 0.1 mM non-essential amino acids, 50 µM β-mercaptoethanol, 50 µg/ml streptomycin, and 50 IU/ml penicillin (all from Gibco-Invitrogen). Supernatants of these cell cultures were assayed for IL-6 (eBisocience), IL-12p40 subunit (R&D systems) by ELISA.

### Statistical Analysis

Data are presented as means ± SD. The differences between mean values were estimated using an ANOVA test followed by pairwise Student's t tests. A P value of less than 0.05 was considered significant (*P<.05, **P<.01, and ***P<.001). All experiments were repeated at least 3 times.

## Results

### Adoptive Transfer of in vivo Instructed Lung DCs is Sufficient to Induce Allergic Sensitization

**Figure 1:**
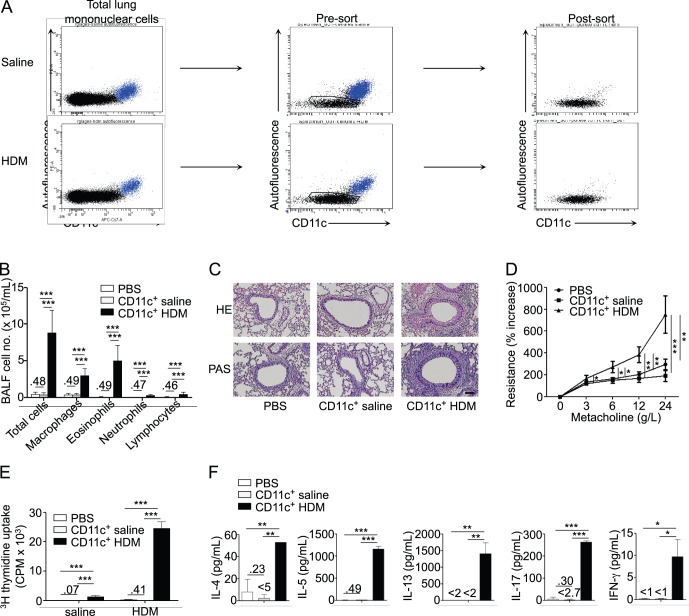
Allergic airway sensitization by adoptive transfer of lung DCs. Naive mice were transferred i.t. with FACS-sorted lung CD11c^+^ DCs from saline- or HDM-treated donors, challenged 10 and 11 d later with HDM and sacrificed 3 d later (n = 6 recipient mice per group). A, Gating strategy and post-sort phenotype of lung DCs from saline- and HDM-treated donor mice. Alveolar macrophages (in blue) were excluded based on their autofluorescence and CD11c^hi^ phenotype. B, Total and differential cell counts in BAL fluid. C, Hematoxylin and eosin (HE, upper panels) and periodic acid-Schiff (PAS, lower panels) staining of lung sections (scale bar: 100 µm). D, Measurement of dynamic airway resistance. E, Proliferation of BLN cells after *in vitro* HDM stimulation. F, ELISA measurement of IL-4, IL-5, IL-13, IL-17 and IFN-γ in the supernatant of HDM-stimulated BLN cells.

We first aimed at developing the most physiological possible model of allergic airway sensitization based on the adoptive transfer of lung DCs. Mice repeatedly exposed to i.n. HDM develop an airway disease that resembles human airway allergy [22], [23]. We reasoned that lung DCs of HDM-treated mice should be able to take up and process HDM antigens *in vivo* and, upon i.t. transfer to naïve recipient mice, to induce HDM-specific T_H_2 cell differentiation and airway allergy. We thus treated donor C57Bl/6 mice with a single i.n. injection of HDM. One day later, we purified CD11c^+^ lung DCs, defined classically as CD11c^+^ non-autofluorescent cells (**Figure 1a**), and transferred them directly by i.t. instillation to naïve recipient syngeneic mice. Upon subsequent exposure to a low dose of HDM, mice that received lung DCs from HDM-treated mice, but not mice that received lung DCs from saline-treated mice, developed signs of airway allergy. These included increased inflammatory cell infiltration (**Figure 1b–c**) and mucus production in the airways (**Figure 1c**), as well as AHR (**Figure 1d**).

When assessing the profile of the adaptive response induced by the transfer of lung DCs from HDM-treated mice, we observed that restimulation of BLN cells of recipient mice with HDM led to significant cell proliferation (**Figure 1e**) and production of the T_H_2 cytokines IL-4, IL-5 and IL-13 (**Figure 1f**). We also detected a significant production of IL-17, and a low but significant production of IFN-γ (**Figure 1f**), additional features of HDM-induced airway allergy in mice [22], [24].

**Figure 2:**
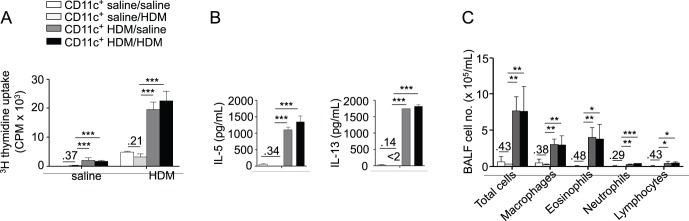
In vivo exposure to HDM is required for obtaining lung DCs with proallergic activity. Lung DCs from saline- or HDM-treated mice (saline/or HDM/) were isolated and cultured in the presence or absence of HDM (/saline or/HDM). Cultured cells were then transferred to recipient mice that were challenged with HDM and sacrificed as in Figure 1 (n = 6 recipient mice per group). A, Proliferation of BLN cells after *in vitro* HDM stimulation. B, ELISA measurement of IL-5 and IL-13 in the supernatant of HDM-stimulated BLN cells. C, Total and differential cell counts in BAL fluid.

Accumulating evidence indicates that allergen-induced activation of structural cells is essential for the promotion of a pro-T_H_2 phenotype in DCs [25], [26], although activation of innate immune signaling within lung DCs also is required [22]. Furthermore, the lung DC pool is modified following allergen exposure, and de novo recruited inflammatory DCs were proposed to play a preferential role in the activation of T_H_2 cells [5], [10]. We thus next aimed at testing whether lung DCs require *in vivo* exposure of the lung to HDM or whether direct HDM stimulation of steady-state lung DCs *ex vivo* may suffice for them to acquire pro-T_H_2 activity. We observed that DCs isolated from the lungs of HDM-treated mice, but not DCs exposed to HDM only *in vitro*, were able to induce HDM-specific adaptive type 2 responses (**Figure 2a–b**) and eosinophilic airway inflammation (**Figure 2c**) following transfer in naïve recipient mice. Thus, lung DCs may induce allergic airway sensitization upon adoptive transfer to naïve recipients only if they originate from allergen-exposed donors.

### Lung CD11b^+^ DCs, but not CD11b^−^CD103^+^ DCs, Induce Allergic Airway Sensitization

**Figure 3:**
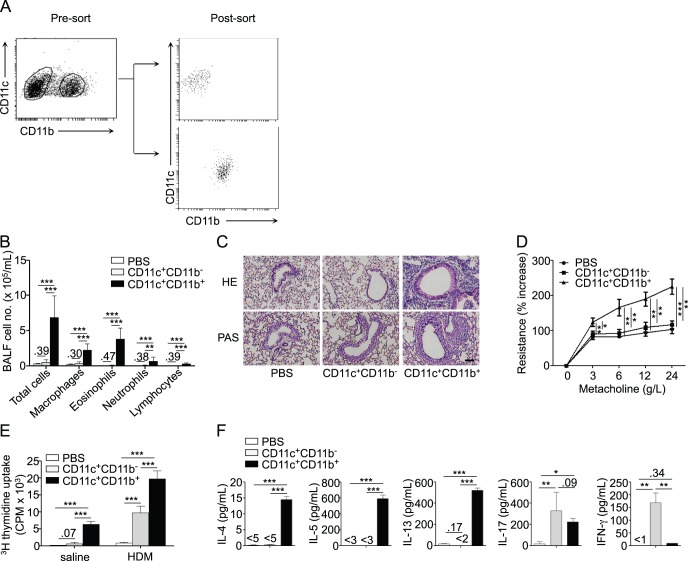
CD11b^+^, but not CD11b^−^, lung DCs promote allergic responses following i.t. transfer. CD11b^+^ and CD11b^−^ lung DCs from HDM-treated mice were isolated and transferred to naïve recipients. Recipients were HDM-challenged and sacrificed as in Figure 1 (n = 6 recipient mice per group). A, Gating strategy and post-sort phenotype of lung CD11b^+^ and CD11b^−^ DCs from HDM-treated donor mice. B, Total and differential cell counts in BAL fluid. C, Hematoxylin and eosin (HE, upper panels) and periodic acid-Schiff (PAS, lower panels) staining of lung sections (scale bar: 100 µm). D, Measurement of dynamic airway resistance. E, Proliferation of BLN cells after *in vitro* HDM stimulation. F, ELISA measurement of IL-4, IL-5, IL-13, IL-17 and IFN-γ in the supernatant of HDM-stimulated BLN cells.

Using the model established above, we next aimed at systematically determining the respective contribution of the different known lung DC subsets to the development of airway allergy in response to HDM. Lung DCs may be first grossly divided in 2 populations based on their differential expression of CD11b [8], [9]. Lung CD11c^+^CD11b^−^ and CD11c^+^CD11b^+^ DCs were sorted from the lungs of HDM-treated mice (**Figure 3a**), and directly transferred to naïve recipient mice, which were later challenged with i.n. HDM. We observed that mice transferred with lung CD11c^+^CD11b^−^ DCs did not develop significant signs of airway allergy when compared with control mice that received PBS only (**Figure 3b–c**). In contrast, mice that received lung CD11c^+^CD11b^+^ DCs from HDM-treated donors developed significant airway eosinophilia (**Figure 3b–c**), goblet cell hyperplasia (**Figure 3c**) and AHR (**Figure 3d**).

In vitro HDM restimulation of BLN cells from mice transferred with lung CD11c^+^CD11b^+^ DCs indicated that these cells efficiently primed HDM-specific type 2 responses (**Figure 3e–f**). Significant production of IL-17 and minimal secretion of IFN-γ were also observed in the BLN cell supernatant (**Figure 3f**). Intriguingly, although CD11c^+^CD11b^−^ DC transfer did not lead to airway inflammation upon HDM challenge of recipient mice, BLN cells from these mice proliferated significantly upon HDM stimulation *in vitro*, and secreted significant amounts of IL-17 and IFN-γ, but not of type 2 cytokines (**Figure 3f**).

**Figure 4:**
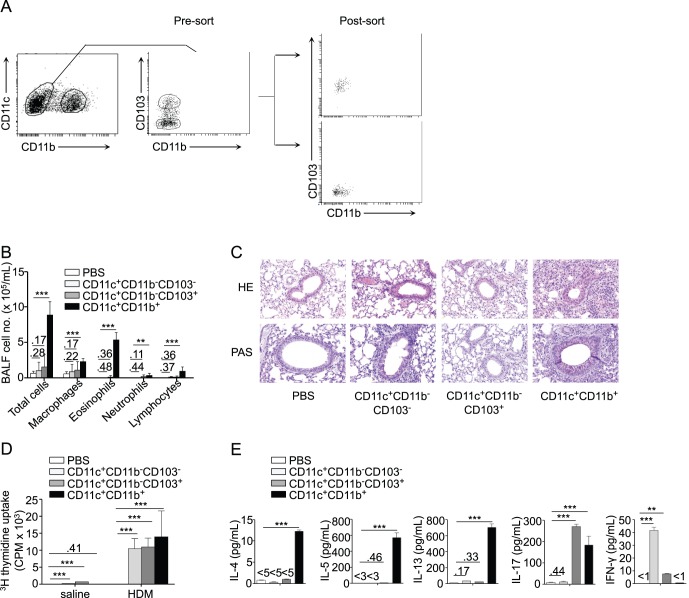
CD11b^−^CD103^+^ lung DCs induce an antigen-specific IL-17 response. Lung CD11b^−^CD103^+^ and CD11b^−^CD103^−^ cells from HDM-treated mice were isolated and transferred to naïve recipients. Recipients were HDM-challenged and sacrificed as in Figure 1. HDM-challenged mice that received lung CD11b^+^ DCs were used as positive controls (n = 6 recipient mice per group). A, Gating strategy and post-sort phenotype of lung CD11b^−^CD103^+^ and CD11b^−^CD103^−^ cells from HDM-treated donor mice. B, Total and differential cell counts in BAL fluid. C, Hematoxylin and eosin (HE, upper panels) and periodic acid-Schiff (PAS, lower panels) staining of lung sections (scale bar: 100 µm). D, Proliferation of BLN cells after *in vitro* HDM stimulation. E, ELISA measurement of IL-4, IL-5, IL-13, IL-17 and IFN-γ in the supernatant of HDM-stimulated BLN cells.

Because the CD11c^+^CD11b^−^ population contained CD103^+^ and CD103^−^ cells (**Figure 4a**), we also studied the consequences of a separate transfer of these two populations on the response of naïve recipient mice to allergen restimulation. Again, none of these two populations induced parameters of airway allergy (**Figure 4b–c**). Yet, upon *in vitro* antigenic restimulation, lymph node cells from mice transferred with both populations exhibited significant proliferative responses (**Figure 4d**). Interestingly, lymph node cells from mice that received CD11b^−^CD103^+^ cells secreted high levels of IL-17 and comparatively less IFN-γ, whereas BLN cells from mice transferred with CD11b^−^CD103^−^ cells secreted IFN-γ only (**Figure 4e**).

### CD11b^+^Ly6C^−^ DCs, but not CD11b^+^Ly6C^+^ DCs Induce Allergic Airway Sensitization

**Figure 5:**
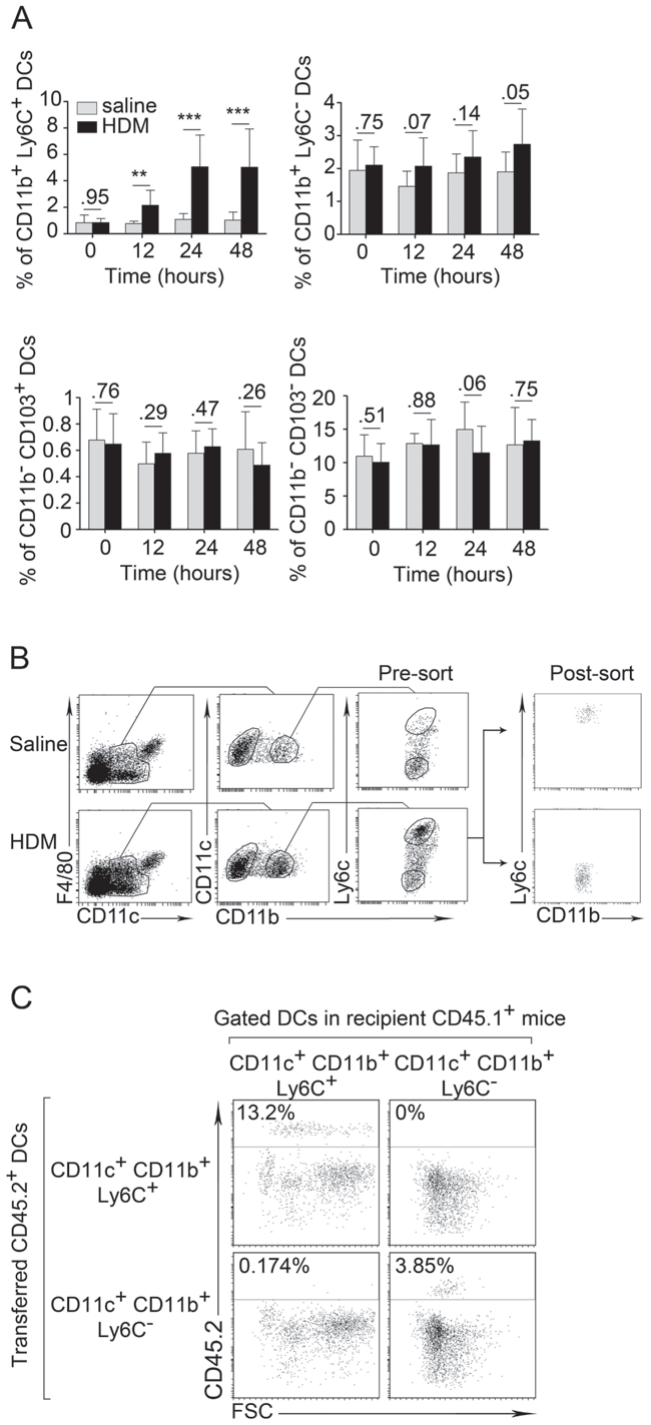
Study of the dynamics of lung DC subsets. A, Percentage of CD11b^+^Ly6C^+^, CD11b^+^Ly6C^−^, CD11b^−^CD103^+^ and CD11b^−^CD103^−^ DCs among total mononuclear lung cells at different time points following i.n. injection of either saline or HDM to naïve mice (n = 4 mice per group). B, Gating strategy and post-sort phenotype of CD11b^+^ lung DCs based on their expression of Ly6C after i.n. injection of saline or HDM. C, Analysis of the percentage of CD45.2^+^ DCs among CD11b^+^Ly6C^+^ and CD11b^+^Ly6C^−^ lung DCs in CD45.1 mice transferred 24 h earlier with either CD11b^+^Ly6C^+^ or CD11b^+^Ly6C^−^ lung DCs from CD45.2 mice. All these analyses were performed using flow cytometry.

The pool of lung CD11b^+^ DCs expands following allergenic challenge of the airways [10], [27], which correlates with the recruitment of blood Ly6C^+^ monocytes [25]. In different contexts, including acute lung inflammation, Ly6C^+^ monocytes were shown to differentiate into inflammatory DCs [5], [12], [28]–[30], which may retain expression of the Ly6C marker as a remnant of their origin [12], [30]. Fitting with the possibility that Ly6C^+^ monocytes may differentiate into inflammatory DCs also in allergen-exposed lung, lung FCεRI^+^ DCs with proposed pro-allergic activity express Ly6C [5]. We thus hypothesized that Ly6C could be used as a marker to discriminate inflammatory CD11b^+^ DCs, whose numbers increase following HDM exposure, from resident CD11b^+^ DCs, whose numbers remain stable (**Figure 5a–b**). Numbers of DCs of the CD11b^−^ subsets did not vary significantly in the lung following HDM challenge (**Figure 5a**).

Because Ly6C has been proposed to be a dynamic marker in other tissues, we first aimed at determining whether lung CD11b^+^Ly6C^+^ DCs may differentiate into CD11b^+^Ly6C^−^ DCs, or vice-versa, upon HDM exposure. We thus transferred CD11b^+^Ly6C^+^ or CD11b^+^Ly6C^−^ DCs isolated from the lung of HDM-treated CD45.2^+^ C57Bl6 mice i.t. into HDM-treated CD45.1^+^ C57Bl6 recipients and assessed for their expression of Ly6C the next day. This experiment revealed that the expression of Ly6C is stable on CD11b^+^Ly6C^+^ and CD11b^+^Ly6C^−^ DCs (**Figure 5c**). Ly6C expression was also stable following *in vitro* culture of CD11b^+^Ly6C^+^ and CD11b^+^Ly6C^−^ DCs in the presence or absence of HDM (data not shown). They thus likely represent distinct DC subsets in the lung.

**Figure 6:**
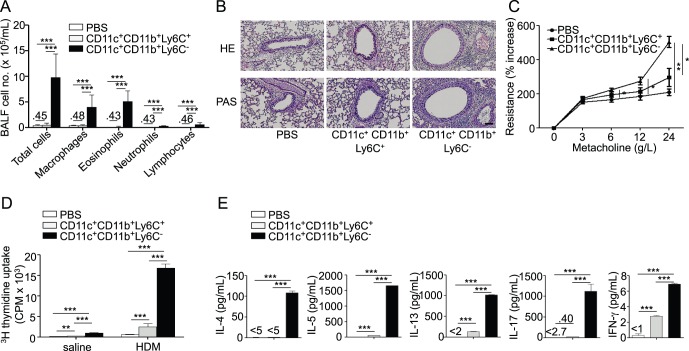
Only resident CD11b^+^Ly6C^−^ lung DCs are capable of inducing airway allergy. CD11b^+^Ly6C^+^ or CD11b^+^Ly6C^−^ lung DCs sorted as in Figure 5B were transferred from HDM-treated mice into naïve recipients. Recipients were HDM-challenged and sacrificed as in Figure 1 (n = 6 recipient mice per group). A, Total and differential cell counts in BAL fluid. B, Hematoxylin and eosin (HE, upper panels) and periodic acid-Schiff (PAS, lower panels) staining of lung sections. C, Measurement of dynamic airway resistance. D, Proliferation of BLN cells after *in vitro* HDM stimulation. E, ELISA measurement of IL-4, IL-5, IL-13, IL-17 and IFN-γ in the supernatant of HDM-stimulated BLN cells.

We observed that, counter-intuitively, only resident CD11b^+^Ly6C^−^ DCs were able to transfer airway allergy to naïve recipient mice (**Figure 6a–c**) and to promote type 2 cytokine, IL-17 and, to a lesser extent, IFN-γ production from BLN cells (**Figure 6d–e**). Transfer of CD11b^+^Ly6C^+^ DCs in contrast only induced negligible BLN cell proliferation and production of IL-5, IL-13 and IFN-γ (**Figure 6d–e**).

Of note, of all 4 DC subsets, only CD11b^+^Ly6C^−^ cells remained able to induce allergic airway sensitization to HDM in naïve recipient mice if individual DC subsets were isolated from the lung of mice 12 h after HDM treatment, instead of 24 h post-treatment as in all figures presented above (data not shown). In addition, similar results were also obtained if, instead of 1×10^6^ cells of each DC subset, naïve recipient mice received numbers of cells of each subset similar to those observed in the mouse lung (i.e. 6×10^5^ CD11b^−^CD103^−^, 3×10^4^ CD11b^−^CD103^+^, 1×10^5^ CD11b^+^Ly6C^−^ or 2,5×10^5^ CD11b^+^Ly6C^+^ DCs) (data not shown).

### CD11c^+^CD11b^+^Ly6C^−^ Lung DCs Present Aeorantigens to Lymph Node T cells

**Figure 7:**
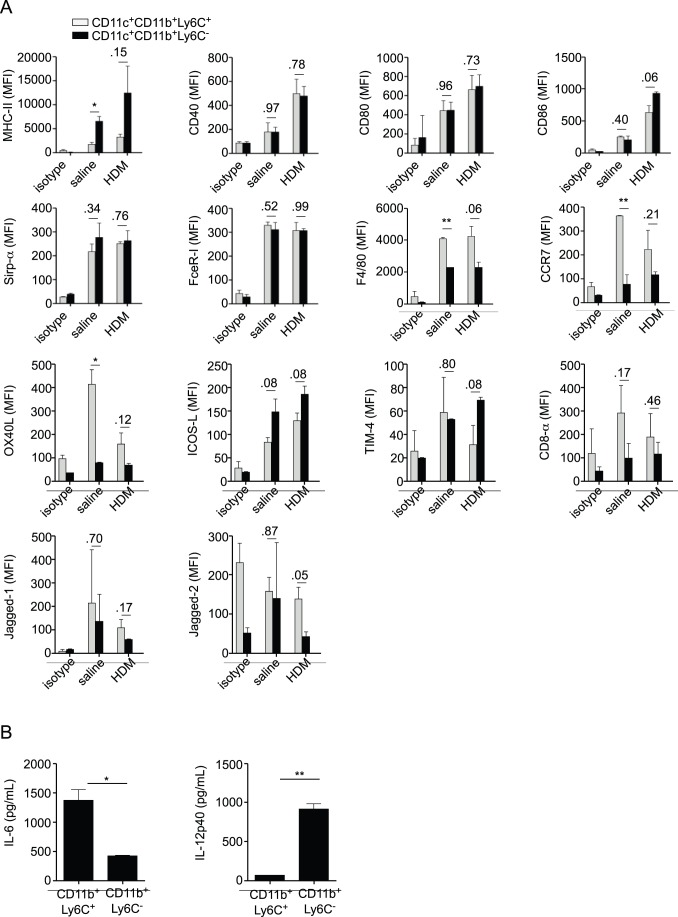
Phenotypic characterization of CD11b^+^Ly6C^−^ and CD11b^+^Ly6C^+^ lung DCs. A, Flow cytometric assessment of the expression levels of MHC-II, CD40, CD80, CD86, Sirp-α, FcεR-I, F4/80, CCR7, OX40L, ICOS-L, TIM-4, CD8α, Jagged-1 and Jagged-2 on the surface of CD11b^+^Ly6C^+^ and CD11b^+^Ly6C^−^ lung DCs from saline- and HDM-treated mice (n = 4 mice per group) (MFI, mean fluorescence intensity). B, ELISA measurement of the production of IL-6 and IL-12p40 by CD11b^+^Ly6C^+^ and CD11b^+^Ly6C^−^ lung DCs from HDM-treated mice.

**Figure 8:**
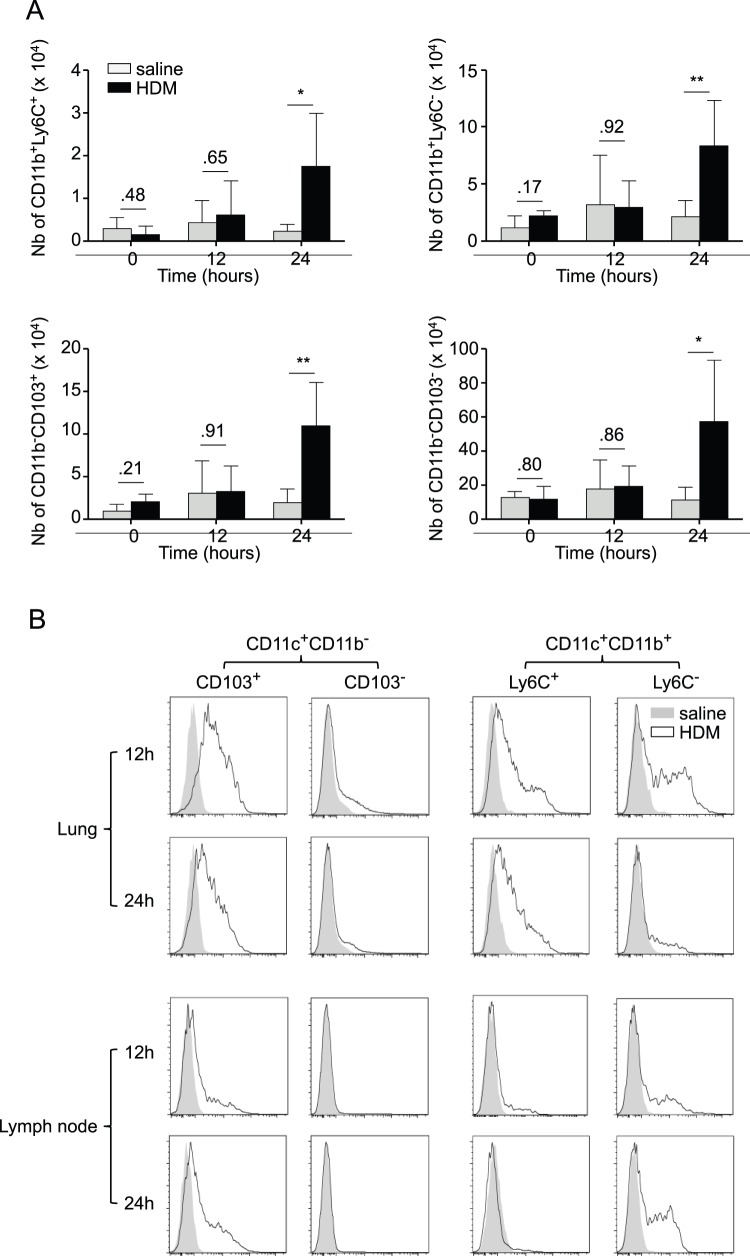
CD11b^+^Ly6C^−^, but not CD11b^+^Ly6C^+^ lung DCs efficiently transport antigen to the BLNs. A, Kinetics of the number of CD11b^+^Ly6C^+^, CD11b^+^Ly6C^−^, CD11b^−^CD103^+^ and CD11b^−^CD103^−^ DCs in the bronchial lymph node following i.n. injection of either saline or HDM to naïve mice (n = 4 mice per group). B, Representative flow cytometric analysis of the presence of antigen-loaded (OVA-FITC^+^) CD11b^+^Ly6C^+^, CD11b^+^Ly6C^−^, CD11b^−^CD103^+^ and CD11b^−^CD103^−^ DCs in the lungs and BLNs of naïve mice injected i.t. 12 or 24 h earlier with either saline or HDM combined with OVA-FITC.

We finally aimed at understanding the differences in the biology of CD11b^+^Ly6C^−^ and CD11b^+^Ly6C^+^ DCs that could explain why only the first were able to initiate airway allergy. To prime naïve T cells in the lung draining lymph nodes, lung DCs should be able to mature, and to transport antigens to the lymph nodes. We thus first compared the surface expression of markers whose expression levels indicate DC maturation and activation, along with other phenotypic surface markers, on CD11b^+^Ly6C^−^ and CD11b^+^Ly6C^+^ DCs in the lung of saline or HDM-treated mice. We observed that both subsets expressed similar levels of all markers tested (**Figure 7a**). Of note, they expressed similar levels of FcεRI and Sirp-α (**Figure 7a**), but were negative for c-Kit (data not shown), which were all recently proposed to be markers of pro-T_H_2 DCs in airway allergy [5], [10], [31]. Both subsets also expressed CCR7 (**Figure 7a**). Second, we assessed the production of immunomodulatory cytokines by CD11b^+^Ly6C^−^ and CD11b^+^Ly6C^+^ DCs and observed a preferential production of IL-6 by the CD11b^+^Ly6C^+^ subset and of the IL12 p40 subunit by CD11b^+^Ly6C^−^ DCs (**Figure 7b**). Finally, we studied the dynamics of the distinct DC subsets in the BLNs of HDM-treated mice as well as their respective antigen uptake and delivery to the BLNs by means of i.t. administration of HDM combined with OVA-FITC, as described previously [5], [28]–[30]. We observed that the numbers of all the DC subsets in the BLNs increased following HDM treatment (**Figure 8a**) and that all were able to take up antigen in the lung with various efficiencies (**Figure 8b**). Although both CD11b^+^Ly6C^−^ and CD11b^+^Ly6C^+^ DCs were able to take up antigens in the lung, with CD11b^+^Ly6C^+^ DCs being most efficient, CD11b^+^Ly6C^−^ DCs were clearly superior at transporting them efficiently to the BLNs following HDM exposure (**Figure 8b**).

**Figure 9:**
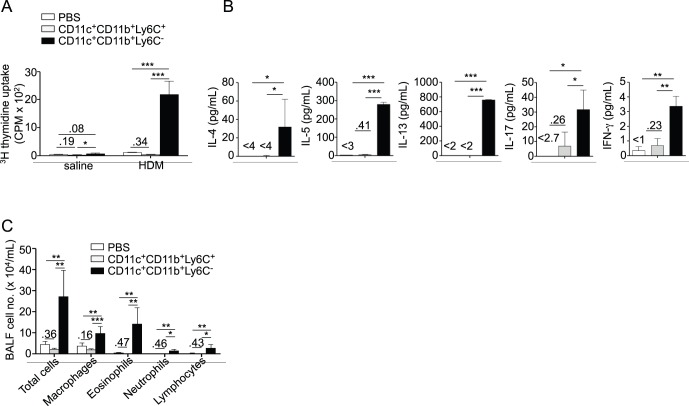
CD11b^+^Ly6C^−^ lung DCs migrating to the lymph nodes have pro-T_H_2 properties. CD11b^+^Ly6C^+^ or CD11b^+^Ly6C^−^ DCs isolated from the BLNs of HDM-treated mice were transferred i.p. into naive recipients (n = 6 recipient mice per group). Recipients were challenged and sacrificed as in Figure 1. A, Proliferation of BLN cells after *in vitro* HDM stimulation. B, ELISA measurement of IL-4, IL-5, IL-13, IL-17 and IFN-γ in the supernatant of HDM-stimulated BLN cells. C, Total and differential cell counts in BAL fluid.

Finally, further supporting that CD11b^+^ Ly6C^−^ DCs indeed are responsible for the activation of naïve T cells in the lymph nodes, we observed that CD11b^+^Ly6C^−^ DCs isolated from the BLNs of HDM-treated donor mice were able to induce T_H_2 cell differentiation (**Figure 9a–b**) and subsequent airway allergy (**Figure 9c**) when transferred i.p. to naïve recipient mice. In contrast, CD11b^+^Ly6C^+^ DCs isolated in the same conditions failed to do so (**Figure 9a–c**).

## Discussion

In this study, we established a model of airway allergy based on the adoptive transfer of lung DCs isolated from donor mice exposed to the clinically relevant allergen HDM. Because this model relies on the *in vivo* instruction and antigen uptake of donor lung DCs, it avoids potential alterations due to *in vitro* culture or allergen loading inherent to most previous adoptive transfer studies. Further supporting the physiological relevance of such an approach, we observed that only DCs isolated from HDM-treated lung, but not lung DCs exposed *ex vivo* to HDM, could transfer allergic airway sensitization to naïve recipient mice. This observation is in line with the idea that lung DCs require instruction from the local allergen-induced inflammatory environment to acquire a pro-T_H_2 phenotype [26], [32]. The fact that de novo recruited inflammatory CD11b^+^Ly6C^+^ DCs are devoid of autonomous pro-allergic activity in our model suggests that resident lung DCs are the essential targets of this instruction. Another as yet untested possibility might also be that CD11b^+^Ly6C^−^ DCs recruited *de novo* following allergen exposure of the lung are involved in allergic airway sensitization.

The recognition of their heterogeneity led to the interesting possibility that, like lymph node DCs [33], distinct lung DC subsets are endowed with specific activities as antigen presenting cells [34], [35]. This notion is however so far only supported by few direct examples. For instance, in models of influenza infection, CD11b^−^CD103^+^ DCs participate in the induction of CD4^+^ and CD8^+^ T cell responses, whereas CD11b^+^ DCs seem unable to activate naïve T cells, and could rather participate in the recruitment of effector cells [18]. The recent studies that addressed the question of whether such a distribution of roles also applies to the induction of T_H_2 responses to inhaled allergens *in vivo* reached contradictory conclusions [5], [13]. We foresee that a possible explanation for these discrepancies lies in the fact that none of these studies examined the activity of each lung DC subset in one unique experimental setting *in vivo*. This indeed obviously complicates the assessment of the relative contribution of each subset to allergic airway sensitization. In this study, we compared the pro-allergic activity of each DC subset systematically in a unique model.

We observed that only resident CD11b^+^Ly6C^−^ DCs are able to induce T_H_2 cell differentiation and subsequent airway allergy to HDM. This raises the question of what the role of the other lung DC subsets in this context might be. Unexpectedly indeed, in spite of their significant recruitment and antigen uptake following HDM exposure, we did not observe any significant autonomous capacity of CD11b^+^Ly6C^+^ inflammatory DCs to prime adaptive immune responses in our model. Instead, allergen-loaded CD11b^+^Ly6C^+^ DCs appear to mostly remain in the lung following HDM exposure, unlike CD11b^+^Ly6C^−^ DCs, which display efficient antigen transport activity to the BLNs. The reason for this differential behavior remains unclear, since both subsets express CCR7. We hypothesize that CD11b^+^Ly6C^+^ DCs may be implicated more in the establishment of an inflammatory environment upon allergenic exposure, or in the recruitment and activation of effector cells. In models of airway allergy focusing on the effector phase of the disease indeed, CD11b^+^ cells were shown to be preferential sources of chemoattractants toward T_H_2 cells and eosinophils [36], [37]. Another study also reported that CD11c^+^CD11b^+^Ly6C^+^ lung DCs may retain inhaled antigens for long periods in the lung and locally reactivate antigen-specific T cells [38], in line with our observation that these cells efficiently take up antigens inhaled in an allergenic context. Very recently, intravital imaging revealed that a fraction of CD11b^+^ DCs is retained in close proximity of the conducting airways upon allergen reexposure in sensitized mice, and that they may locally interact with and activate memory T cells [39]. Whether these cells correspond to CD11c^+^CD11b^+^Ly6C^+^ DCs is a likely possibility that would require further testing.

We also observed that transfer of CD11b^−^CD103^+^ DCs, although devoid of pro-T_H_2 activity, induced HDM-specific responses characterized mostly by the production of IL-17 and, to a lesser extent, of IFN-γ upon transfer *in vivo*. The physiological relevance of this observation is currently unclear, but suggests that this subset may be involved in the induction of previously described non-T_H_2 components of the response to HDM [22], [24]. Interestingly, these cells were also recently shown to be able to reactivate allergen-specific T_H_2 cells in response to LPS stimulation [40]. Thus, unlike in the sensitization phase, where CD11b^+^Ly6C^−^ DCs appear as the essential triggers of T_H_2 cell activation, it is likely that different lung DC subsets may participate in T_H_2 cell reactivation in sensitized individuals [38], [40].

In conclusion, this study supports that, among lung DCs, resident CD11b^+^Ly6C^−^ DCs are the essential triggers of T_H_2 cell activation and allergic airway sensitization to HDM.
